# Sex Differences in Odor Hedonic Perception: An Overview

**DOI:** 10.3389/fnins.2021.764520

**Published:** 2021-10-18

**Authors:** Charlotte Bontempi, Laurence Jacquot, Gérard Brand

**Affiliations:** ^1^Laboratoire de Recherches Intégratives en Neurosciences et Psychologie Cognitive – UR481, Université de Franche-Comté, Besançon, France; ^2^CSGA Centre des Sciences du Goût et de l’Alimentation, Dijon, France

**Keywords:** olfaction, sex differences, hedonicity, pleasantness rating, emotion

## Abstract

Odor hedonic evaluation (pleasant/unpleasant) is considered as the first and one of the most prominent dimension in odor perception. While sex differences in human olfaction have been extensively explored, gender effect in hedonic perception appears to be less considered. However, a number of studies have included comparisons between men and women, using different types of measurements (psychophysical, psychophysiological,…). This overview presents experimental works with non-specific and body odors separately presented as well as experimental studies comparing healthy participants *vs* patients with psychiatric disorders. Contrary to sensitivity, identification or discrimination, the overall literature tends to prove that no so clear differences occur in odor hedonic judgment between men and women. On the whole, gender effect appears more marked for body than non-specific odors and is almost never reported in psychiatric diseases. These findings are discussed in relation to the processes classically implied in pleasantness rating and emotional processes.

## Introduction

Chemical senses are crucial in the animal kingdom and are involved in a large variety of adaptative behaviors such as food search, predators avoidance or mates selection. In humans, the processes implied in olfactory perception are well described while the role and the perceptual characteristics of olfaction are currently discussed (Brand, 2020).

The multidimensional aspect of olfaction is related to the three main perceptual characteristics of odors : the intensity (mainly driven by the odorant concentration), the quality (mainly related to the odorant chemical structure) and the hedonic valence corresponding to the pleasant or unpleasant character of the odor. Among them, odor hedonic perception is universally considered as the first and one of the most salient dimension of olfactory perception since a long time (e.g., Moncrieff, 1966; Schiffman, 1974; Land, 1979). Different aspects are usually considered in studies focusing on odor hedonic perception: the odor valence that refers to the pleasant/unpleasant character of odor stimulus, the liking that corresponds to the perceived pleasantness, the wanting that corresponds to the wish to be further exposed to the same odor stimulus and the emotional response that refers to the influence of odor on psychological and physiological states.

Nonetheless, several questions remain unclear such as the relation between the hedonic space of odors and the molecular properties of odorants. Moreover, methodological questions regarding the hedonic assessment of odors make data interpretation difficult and the cortical treatment of odor pleasantness appears highly complex. Overall, studies on odor hedonic perception reveal a large intra- and inter- subject variability and thus an extreme flexibility of odor hedonic perception. In sensory hedonic perception, gender is usually considered as a major source of variability and sex differences have been reported in the somatosensory modality (e.g., Jönsson et al., 2017; Novembre et al., 2021), in the gustatory modality (e.g., Cristovam et al., 2000) or in the visual modality (e.g., Sorokowski et al., 2014).

With respect to olfaction, sex differences are well-known and have been largely investigated within different processes such as sensitivity, identification, discrimination or memory in healthy subjects (Brand and Millot, 2002; Doty and Cameron, 2009; Sorokowski et al., 2019) and in patients with neurodegenerative disorders (Melis et al., 2019; Solla et al., 2020). Surprisingly, gender as an influencing factor of hedonic responses to odorants is poorly documented. Because numerous studies considered male and female groups, this paper aims to propose a general overview concerning sex differences in odor hedonic perception. For the sake of clarification, non-specific odors and body odors are separately considered. In the same way, type of task (scale, ranking,…), type of recording (electrophysiological, fMRI,…) and properties of odorant (intensity, pungency,…) are also considered. Finally, characteristics of subjects are taken into account such as mood or psychiatric disorders (depression, schizophrenia,…). The observed differences in odor hedonic perception are discussed in relation to the processes classically implied in pleasantness rating and emotional treatment and could serve usefully in future experimental studies using odors as affective or emotional inducers.

## Sex Differences in Healthy Populations: Non-Specific Odors

Data from the literature are reported in two tables, when sex-related differences are demonstrated (Table 1) and when no difference are reported (Table 2). Hedonic judgment is usually evaluated using psychometric (rating scales) and psychophysiological recordings such as electrocardiography (ECG), electroencephalography (EEG), skin conductance response (SCR), or event-related potentials (ERPs), which are sometimes associated with imaging methods. When demonstrated, sex differences in odor hedonic perception seem to be odorant-dependent, albeit not systematic.

**TABLE 1:** Summary of studies in healthy populations showing sex differences in odor hedonic perception (non-specific odors and body odors).

Authors	Number of subjects (Male/Female)	Number and type of odors	Methods	Results
Kenneth, 1924	Data not available	Non-specific odors	Data not available	Women judge camphor, menthol, and ferric valerian odors to being more pleasant than men, whereas the opposite is found for pine oil, cedarwood oil, musk and tonka beans.
Doty et al., 1975	37/41 adults	Body odors	Magnitude estimation	Women judge vaginal secretions odors to being more unpleasant than men.
Doty et al., 1982	5/5 adults	33 body odors	Rating scale from “very unpleasant” to “very pleasant”	Women evaluate breath odors as less pleasant than men.
Wysocki and Gilbert, 1989	43,6%/56,40% adults	4 non-specific odors	5-point scale from unpleasant to pleasant	Women rate eugenol and rose as more pleasant than men while the reverse is noted for isoamyl acetate and mercaptan.
Haidt et al., 1994	71/111 students 58/68 students 109/61 students 28/18 students	Not relevant	32-item Disgust Scale	Women score higher than men.
Broman and Nordin, 2000	Data not available	1 non-specific odor	Aversive detection thresholds	Women present an odor aversion at lower concentrations (*i.e.*, lower aversive detection threshold) than men, and they evaluate concentrations above this threshold as more unpleasant.
Platek et al., 2001	18/32 adults	50 body odors	Visual analogue scale from 0 (not at all pleasant) to 100 (extremely pleasant)	Women assess their own axillary odors as less pleasant than men.
Royet et al., 2003	14/14 adults	126 non-specific odors	functional Magnetic Resonance Imaging	Between resting and olfactory conditions, men demonstrate cerebral activations in the bilateral insula and in the left piriform-amygdala region. In women activations are located in the same areas as well as in the left orbitofrontal cortex.
Stevenson and Repacholi, 2003	23/26 children 19/16 teenagers 15/15 adults	4 body odors 1 non-specific odor	5-point scale with an added visual component from “I dislike it a lot” to “I like it a lot.”	In children, boys assess female sweat odor as more unpleasant than girls. In teenagers, girls rate male sweat odor to being more unpleasant than boys, and caramel odor as more pleasant than boys. In adults, women rate both male and female body odors as more unpleasant than men.
Olofsson and Nordin, 2004	17/19 adults	1 non-specific odor	Category ratio -10 scale Event-related potentials	Women give highest unpleasantness ratings, particularly for the highest odorant (*i.e.*, pyridine) concentration. The amplitude and the latency of P2/P3 component at Cz position are, respectively, larger and shorter in women than in men.
Nordin et al., 2004	272/323 adults	Not relevant	Chemical Sensitivity Scale (CSS)	CSS scores are higher in women than in men.
Martins et al., 2005	20/20 heterosexual adults 20/20 homosexual adults	24 body odors	Forced-choice preference judgments and strength of preference (10-point scale)	Homosexual men prefer body odors from homosexual men while both heterosexual men and women as well as homosexual women prefer body odors from heterosexual men.
Olatunji et al., 2007	249/744 adults (study 2)	Not relevant	27-items Disgust Scale	Women score higher than men for the three disgust dimensions: Core Disgust, Animal Reminder Disgust, and Contamination-Based Disgust
Seo et al., 2008	53/87 adults	12 non-specific odors	6-points Likert scale ranging from 1 (very pleasant) to 6 (very unpleasant)	Odor hedonic valence is influenced by the presence of a verbal label. Hedonic valence is significantly modified for odors of cinnamon, licorice, rose and clove in women, and for odors of shoe leather, lemon, and pineapple in men.
Thuerauf et al., 2009	86/86 adults	16 non-specific odors	Two bipolar rating scales: one (relative hedonic estimates) ranging from -10 (unpleasant) to +10 (pleasant) and one (absolute hedonic estimates) ranging from 0 to +10	Odor hedonic valence (*i.e*., both relative and absolute hedonic estimates) is always higher in women than in men for both pleasant and unpleasant odors.
Seubert et al., 2009	13/12 adults	3 non-specific odors	7-points Likert scale from -3 (extremely unpleasant) to +3 (extremely pleasant), with 0 indicating a neutral affective value.	Sex differences are only observed for the pleasant vanillin odor. Women rate this odor as more pleasant than do men.
Seo et al., 2009	50/50 adults	6 non-specific odors	Emotional response test: 25 paired semantic differential scales Odor hedonic rating: 9 cm line scale ranging from 0 (extremely dislike) to 9 (extremely like)	Women prefer odors characterized as more “bright” “faint”, “warm”, “common,” and, “intellectual”. Men prefer odors characterized as more “refined”, “familiar” and “manlike”.
Trellakis et al., 2011	17/14 adults	6 non-specific odors	100 mm visual analog scale) (lowest hedonic value 0 mm, highest 100 mm).	For patchouli oil, male subjects showed a significantly higher odor pleasantness than female.
Triscoli et al., 2014	9/9 adults	3 non-specific odors	Visual analog scale from “not at all pleasant” to “very pleasant”	From the 5th presentation, the perceived pleasantness decreases in men while it remains constant in women.
Hoffmann-Hensel and Freiherr, 2016	13/12 right-handers’ adults	4 non-specific odors	Physiological responses (Skin conductance responses and breathing data) Behavioral responses (odor perception reaction times)	Only men show differences related to the pleasantness of odors for skin conductance responses.
Tullio Luizza et al., 2017	285/233	Not relevant	Body odor Disgust Scale	Women report a higher level of body odor disgust than men.
Henkin, 2018	134/178 adults	4 non-specific odors	Scale from 0 to 100 reflecting pleasantness and scale from 0 to -100 reflecting unpleasantness	No sex differences before treatment. After treatment (400 and 600mg of oral theophylline) women assess pyridine and thiophene as more unpleasant than men.
Ferdenzi et al., 2019	20/20 French adults 175/105 Malagasy adults	1 non-specific odor	Scale from 1 (not at all pleasant) from 7 (very pleasant)	Sex differences are only found in French participants. Women rate phenylethyl alcohol as more pleasant than men.

**TABLE 2:** Summary of studies in healthy populations showing no sex differences in odor hedonic perception (non-specific odors and body odors).

Authors	Number of subjects (Male/Female)	Type of odors	Methods	Results
Doty et al., 1978	10/10 adults	10 body odors	Magnitude estimation	No sex differences. However, in both men and women, female axillary odors are judged weaker and less unpleasant than male axillary odors.
Brand and Jacquot, 2001	15/15 right-handed adults	4 non-specific odors	Skin conductance responses	No sex differences.
Bensafi et al., 2002b	10/22 adults	2 non-specific odors	Scale from 0 (not at all pleasant) to 5 (extremely pleasant) Response times	No sex differences.
Bensafi et al., 2002a	8/10 adults	6 non-specific odors	Likert scale from 1 (not at all pleasant) to 9 (extremely pleasant) Heart rate and skin conductance	No sex differences.
Lundström and Hummel, 2006	17/17 adults	1 non-specific odor	Event-related potentials Visual scale ranging from 0 (unpleasant) to 100 (pleasant).	No sex differences in hedonic ratings However, women express larger amplitudes and longer latencies in the left hemisphere, whereas in men this phenomenon occurs in the right hemisphere.
Boesveldt et al., 2010	20/20 adults	4 non-specific odors	Visual analogue scale from “extremely unpleasant” to “extremely pleasant” Reaction times	No sex differences.
Knaapila et al., 2012	153/244 adults	1 body odor	9-point Likert scale ranging from 0 (extremely unpleasant) to 9 (extremely pleasant) through 5 (neither unpleasant, nor pleasant)	No sex differences. However, in women hedonic rating of androstenone is related to sexual intercourse experiences
Ferdenzi et al., 2013	59/151 adults from Geneva 144/207 adults from Liverpool 87/124 adults from Singapore	56 (in Geneva and Liverpool) and 59 (in Singapore) non-specific odors	Visual analog scale labeled from 0 (not at all pleasant) to 200 (extremely pleasant)	No sex differences.
Lübke and Pause, 2014	26/25 adults	1 body odor	Scale from 0 (not at all pleasant) to 10 (extremely pleasant)	No sex differences. However, in men with higher testosterone level, androstenone is perceived as more unpleasant than in men with lower testosterone level. Similarly, in women with higher estradiol level, androstenone is perceived as more unpleasant than in women with lower estradiol level
Mutic et al., 2016	15/16 adults	27 body odors	Visual analog scale from 0 (not pleasant) to 100 (extremely pleasant)	No sex differences. However, women body odor is globally rated as less pleasant than men body odor.
Knaapila et al., 2017	33/93 adults	12 non-specific odors	9-point scale from −4 to +4	No sex differences.
Ferdenzi et al., 2019	20/20 French adults 175/105 Malagasy adults	3 body odors	Scale from 1 (not at all pleasant) to 7 (very pleasant)	No sex differences.

### Psychometric Studies

From the beginning of the 20^*th*^ century, Kenneth (1924) showed that women rated camphor, menthol and ferric valerian as more pleasant than men while the reverse was found for pin oil, cedarwood oil, musk and tonka beans. Later, Wysocki and Gilbert (1989) demonstrated that women rated eugenol and rose as more pleasant than men while the reverse was noted for isoamylacetate and mercaptan. More recently (Seubert et al., 2009), the pleasant vanillin odor was rated as more pleasant by women compared to men whereas no difference was observed for the unpleasant hydrogen sulfide odor as well as for eugenol, a bimodal pleasant/unpleasant odor. In addition, it has been shown that men assessed patchouli oil as more pleasant than women (Trellakis et al., 2011) and women assessed phenyl ethyl alcohol as more pleasant than men (Ferdenzi et al., 2019). Interestingly, in hyposmic populations, no sex difference was observed before treatment whereas after treatment (theophylline) women assessed pyridine and thiphene as more unpleasant than men (Henkin, 2018).

More globally, it seems that women evaluate the pleasantness of perceived odors in a more extreme manner than men for both pleasant and unpleasant polarities (Thuerauf et al., 2009). This observation appears congruent with results obtained from self-rated psychological scales, especially in the case of disgust. For instance, Haidt et al. (1994) used the 32-item Disgust Scale (DS) and showed that women scored higher than men. Factor analyses revealed that DS taps three dimension of disgust: core disgust, animal reminder disgust and contamination-based disgust. It was then demonstrated that women scored higher than men for the three disgust dimensions (Olatunji et al., 2007). Using a specific body odor disgust scale (BODS), Tullio Luizza et al. (2017), revealed a reliable but small effect of gender indicating that women reported a higher level of body odor disgust than men. In another way (Nordin et al., 2004), the CSS scores (Chemical Sensitivity Scale used to quantify affective reactions to odorous/pungent substances in the environment) were found to be higher in women than in men. However, studies carried out in different countries (Ferdenzi et al., 2013; Knaapila et al., 2017) found no sex-related differences in odor hedonic perception whatever the odorants and the country. These discrepancies may be due to several factors, especially the experimental conditions (i.e., type and number of odorants, type of rating scales,…).

Concerning the odorant properties, the perceived intensity, which is related to the stimulus concentration, is an important determinant of hedonic estimation and can reveal gender differences. For instance, women rated several concentrations of pyridine as more unpleasant than men (Broman and Nordin, 2000). These findings were later confirmed with three pyridine concentrations (Olofsson and Nordin, 2004). Additionally, Croy et al. (2017) showed a significant correlation between olfactory threshold and disgust ratings, but only in men. Furthermore, the experimental context can strongly influence many aspects of olfactory perception such as detection threshold (e.g., Rabin and Cain, 1986) discrimination (e.g., Jehl et al., 1995) and hedonicity (e.g., Djordjevic et al., 2008). First, considering repetitive exposure to three pleasant odorant stimuli (coconut, aloe and flowers), Triscoli et al. (2014) demonstrated that perceived pleasantness decreased from the first five presentations in men whereas it remained stable in women. Interestingly, the reverse occurred for “wanting” ratings (defined as “the wish to be further exposed to the same olfactory stimulus”) that remained stable in men while it decreased during repetitive stimulations in women. Second, it has been shown that verbal labels associated to odors can influence olfactory perception (Herz and von Clef, 2001). Using odorants from the Sniffin’Stick Identification Test (Hummel et al., 1997), a study compared hedonicity before and after the presentation of an odor label (Seo et al., 2008). Results revealed significant changes for specific odors of cinnamon, rose, cloves and licorice in women and for specific odors of shoe leather, pineapple, lemon in men. However, for coffee odor the effect of odor label was observed in both men and women. With an other paradigm, Seo et al. (2009) asked participants to rate the emotional responses of dairy odors using 25 adjective pairs (e.g., common/rare, natural/artificial, usual/unusual, attractive, denial,…) as well as the hedonicity using a line scale. Results showed that both men and women preferred dairy odors that were characterized as more “comfortable,” “attractive” and “fragrant.” However, women preferred odors characterized as more “bright,” “faint,” “warm,” “common” and “intellectual,” while men preferred odors characterized as more “refined,” “familiar” and “manlike.”

Finally, several comparative exposure conditions have not been yet explored. For instance, the hypothesis that gender differences in odor hedonic perception could be related to the nostril stimulated (Brand et al., 2001; Disjkersrhuis et al., 2002) would be worth investigating. Additionally, further studies are needed to compare ortho *versus* retronasal stimulations or to examine the impact of the odorant qualities. For instance, the impact of trigeminal component of odorants (Brand, 2006) has never been explored, except in the study of Wallrabenstein et al. (2015) suggesting that the activation of VN1R1 by hedione might play a role in sex-specific modulation of hormonal secretion in humans. More specifically, because of the importance of pleasantness in food consumption, research should explore the role of food composition in sensory attributes evaluation. As a precursor, the work of Rosa et al. (2020) showed that women exhibited a greater ability than men to detect pleasantness of an odor rich in free fatty acid such as the mullet roes.

### Psychophysiological and Imagery Studies

A number of studies have used psychophysiological measures (SCR, ECG, EEG, or ERPs) in response to pleasant and unpleasant odors (e.g., Alaoui-Ismaïli et al., 1997a; Glass et al., 2014). Independently from gender, results showed a more marked effect with unpleasant odors compared to pleasant odors. Surprisingly, gender differences in odor hedonic perception using psychophysiological measures are poorly documented, contrary to psychometric measurements. Some studies included only women (e.g., He et al., 2014, 2016; Pichon et al., 2015) or did not consider sex differences (e.g., Alaoui-Ismaïli et al., 1997b; Bensafi, 2002; Djordjevic et al., 2008; Ferdenzi et al., 2017). Some other works compared men and women in skin conductance, heart rate responses or reaction time using pleasant and unpleasant odors (Brand and Jacquot, 2001; Bensafi et al., 2002a,b; Boesveldt et al., 2010). Data revealed no gender-related differences according to the hedonic valence of odorants. By contrast, in an other study (Hoffmann-Hensel and Freiherr, 2016), only men showed differences related to the pleasantess of odors (orange, cherry, vomit and spoiled fish) in skin conductance responses. Using ERPs with the bimodal odor of peppermint oil, Lundström and Hummel (2006) demonstrated that, although there were no differences in hedonic ratings between men and women, a sex-related difference occurred in hemispheric responses, i.e., women expressed larger amplitudes and longer latencies in the left hemisphere while the same phenomenon was found in the right hemisphere in men. Another study (Olofsson and Nordin, 2004) conducted with three concentrations of pyridine indicated that the amplitude and the latency of P2/P3 component at Cz position were, respectively, larger and shorter in women than in men.

For several years, research concerning brain activity in olfactory perception, especially in hedonic perception, seeks to determine cerebral areas activation and to understand the underlying mechanisms (Zou et al., 2016) that are always currently discussed (Ruser et al., 2021). However, few studies have considered gender, probably because the number of participants recruited was small. Among them, the study of Royet et al. (2003) reported differential activation in men and in women, particularly in the left orbitofrontal cortex.

## Sex Differences in Healthy Populations: Body Odors

There is evidence that body odors are involved in human communication and contribute to social interactions in daily life. Indeed, body odors carry important information relating to emotional state (de Groot et al., 2015), mate selection and attractiveness (Singh and Bronstad, 2001) as well as hormonal state (Preti et al., 1986, 2003). Body odors derive from volatile compounds present in urine, vaginal secretions or breath and produced by degradation of bacteria on human skin (e.g., axillary zone, feet,…). These odors are mainly composed of aldehydes, ketones and carboxylic acid (Natsch and Emter, 2020). In the current society, body odors are usually perceived as unpleasant although interindividual differences have been reported, particularly between men and women.

In a pioneer study (Doty et al., 1975), vaginal secretions were collected from four women during 15 consecutives menstrual cycle and the odor pleasantness was rated by both men and women. Results clearly showed that women assessed the odors as more unpleasant than men. In the same way (Doty et al., 1978), hedonic valence of male and female axillary odors were rated by men and women and no difference was observed. In contrast (Doty et al., 1982), when both men and women rated male and female breath odors, the breath odor of males were rated as more intense and less pleasant than the breath odor of females. In addition, women gave significant lower pleasantness ratings to the breath odors than men. Interestingly, both men and women assigned the breath odor to the correct gender class and an inverse relation between breath odor intensity and pleasantness was observed. Concerning body odors, usually collected using T-shirt, cotton or gauze pieces placed under armpits, it appears that women assessed their own body odor as less pleasant than did men toward theirs (Platek et al., 2001). A comparative study (Stevenson and Repacholi, 2003), examined the hedonic estimation of body odor from adults by children, teenagers and adults. In children, boys rated women body odors as more unpleasant than girls while in teenagers, girls rated men body odors as more unpleasant than boys. In adults, women rated both male and female body odors as more unpleasant than men.

However, several factors could influence the hedonic estimation of body odors. For instance, the hypothesis that sexual orientation of donor could induce changes in pleasantness rating has been tested (Martins et al., 2005). Results indicated that homosexual men preferred body odors from homosexual men while both heterosexual men and women as well as homosexual women preferred body odors from heterosexual men. In another way (Mutic et al., 2016), a comparative analysis assessed the pleasantness of body odors collected during an intensive physical exercise and showed that women body odor was globally rated as less pleasant than those of men without sex-related differences.

Among body odors, androstenone is probably the most studied in experimental research. Androstenone is a steroid hormone commonly found in sweat and urine of male mammalians. In humans, androstenone is present in axillary regions in greater quantity in men than in women (Gower et al., 1985). Because of a large genetic polymorphism of genes encoding the olfactory receptors (ORs) among human’s population, many people and mostly men cannot detect androstenone (Wysocki and Beauchamp, 1984; Bremmer et al., 2003). Like other smells, the pleasantness of androstenone is context-dependent: perceived as unpleasant when associated with urine and sweat or perceived as pleasant when associated with floral cues (Hasin-Brumstein et al., 2009). Surprisingly, although androstenone is widely studied, few publications have been dedicated to gender differences. Elsewhere, several experiments considered additional specific parameters that are possibly related to androstenone pleasantness. For instance (Lübke and Pause, 2014), in men with higher testosterone level, androstenone was perceived as more unpleasant than in men with lower testosterone level. Similarly, in women with higher estradiol level, androstenone was perceived as more unpleasant than in women with lower estradiol level. In another way, it has been suggested (Knaapila et al., 2012) that specifically in women, hedonic rating of androstenone is related to sexual intercourse experiences. Indeed, androstenone is rated as more pleasant by women who had a sexual intercourse experience with at least one partner, compared to those who reported never had sexual intercourse. Finally, the unique study comparing the hedonic perception of androstenone in relation to gender (Ferdenzi et al., 2019) found no sex differences for any of the perceptual variables, including pleasantness.

## Sex Differences in Psychiatric Disorders

Olfactory deficits have been reported in psychiatric diseases, particularly in schizophrenia, depressive and bipolar disorders (e.g., Atanasova et al., 2008; Turetsky et al., 2009; Lahera et al., 2016; Brand and Schaal, 2017; Kamath et al., 2017; Kiparizoska and Ikuta, 2017). Patients usually presented lower olfactory sensitivity, discrimination and identification scores than healthy people (e.g., Postolache et al., 2002; Chen et al., 2018). Moreover, it has long been shown that in these diseases, olfactory impairment is stage-dependent and treatment-dependent in these diseases as it has been shown for a long time (Gross-Isseroff et al., 1994; Sirota et al., 1999). Olfactory dysfunction could also be disease-specific as suggested in a comparative study between bipolar, depressive and schizophrenic patients (Li et al., 2021). However, some brain areas such as amygdala, hippocampus, insula, anterior cingulate cortex and orbitofrontal cortex, involved in the above-mentioned psychiatric disorders – especially mood disorders – are linked to the olfactory system in relation to odor hedonic perception (e.g., Soudry et al., 2011). This relationship warrants further experimental studies on odor pleasantness ratings.

In schizophrenia, both men and women overevaluated the pleasantness of isoamyl acetate at high concentrations and underrated the lower concentrations compared to healthy subjects (Kamath et al., 2013). By contrast, another study (Walsh-Messinger et al., 2018) showed that schizophrenic men rated pleasant odors (orange, apple, mint,…) less pleasant than healthy men whereas no difference was observed between healthy and schizophrenic women. When studies related to this topic focused on gender differences in patients with schizophrenia, some of them showed that schizophrenic men rated pleasant odorants as more pleasant than schizophrenic women and healthy participants whatever the concentration level (Moberg et al., 2003; Robabeh et al., 2015). Other studies found no gender differences in odor hedonic judgment (Hudry et al., 2002; Lui et al., 2020).

In depression, the disease effect on odor hedonic ratings is not clearly demonstrated (e.g., Lombion-Pouthier et al., 2006; Swiecicki et al., 2009; Atanasova et al., 2010). When gender effect was considered in depression (Clepce et al., 2010), no difference between men and women was found in odor hedonic judgment. Similar data were obtained in bipolar disorder patients (Cummings et al., 2011) although the authors noted that women tended (i.e., in a non-significant way) to rate pleasant and unpleasant odors as more pleasant than men.

## Conclusion

Contrary to sensitivity, identification or discrimination, this overview tends to prove that not such a clear differences occur in odor hedonic estimation between men and women. Gender effect appears more marked for body than for non-specific odors and is almost never reported in psychiatric diseases. It must be noted that no published study has yet focused on gender effect in odor hedonic estimation in neurodegenerative diseases (Alzheimer, Parkinson,…) while olfactory capabilities have been largely assessed (e.g., Doty, 2012; Woodward et al., 2017). When differences are reported, albeit not systematic, it seems that women overrated pleasant odors and underrated unpleasant odors compared to men. In accordance with the view that the hedonic processing of odor stimuli is an emotional rather than an analytical task and because sex differences occur in emotion (Wester et al., 2002), gender should logically affect clearly both the polarity and the extremity of the odor hedonic estimates. Thus, these findings regarding odor hedonic perception are rather surprising insofar as women are considered more responsive to emotional stimuli than men (Lithari et al., 2010) including auditory stimuli such as music and sounds, emotional words or emotional tones of voice (e.g., Schirmer et al., 2002, 2005; Bachorowski and Owren, 2003) visual stimuli such as pictures and films (see Cahill, 2006 for a review) or danger-related stimuli (Williams and Gordon, 2007).

Additionally, except for pyridine and androstenone, there is not enough consistent data to draw conclusions on particular odorants. Moreover, most of studies focus on several parameters and rarely only on hedonic estimation. Thus, the odorants, tasks and participant characteristics are generally heterogeneous from one study to another (Clepce et al., 2014) which induces discrepancies and makes an overall conclusion difficult.

The main hypothesis related to this tenuous difference between men and women probably concerns the great intra- and inter-variability in hedonic responses to odors. Indeed, numerous factors can influence odor perception (Greenberg et al., 2013) and certainly can influence the hedonic estimates and leveled the scores into groups of participants. Inter-individual differences can occur in relation to sensitivity, familiarity, experience and memory toward the odorant stimulus (e.g., Delplanque et al., 2008), as well as in relation to age, culture, eating habits, medication and personality traits. Intra-individual differences can occur in relation to physiological states (e.g., arousal, weariness, stress, hunger,…), hormonal states (e.g., Trellakis et al., 2011) and psychological states (attention, motivation, mood,…). Besides, intra- and inter-individual differences in odor preferences are not specific to humans and have been reported in animals (e.g., Jagetia et al., 2018) in rigorously homogeneous cohorts. This suggests that odor hedonic perception is a highly complex response that prevents direct and relevant comparisons between men and women in the general population. Besides, current research demonstrates that individual pleasantness of odors is not detectable in fMRI (Ruser et al., 2021), probably because this process is coded in joint networks (Mantel et al., 2019). Thus, future research in this field - especially food studies, toxicological or specific exposure studies, quality of life studies, must cross several factors in a multidimensional approaches among which gender could be included in order to contribute to the knowledge of the human affective neurosciences (Becker et al., 2019).

## Author Contributions

All authors listed have made a substantial, direct and intellectual contribution to the work and approved it for publication.

## Conflict of Interest

The authors declare that the research was conducted in the absence of any commercial or financial relationships that could be construed as a potential conflict of interest.

## Publisher’s Note

All claims expressed in this article are solely those of the authors and do not necessarily represent those of their affiliated organizations, or those of the publisher, the editors and the reviewers. Any product that may be evaluated in this article, or claim that may be made by its manufacturer, is not guaranteed or endorsed by the publisher.
